# Anatase TiO_2_-Decorated Graphitic Carbon Nitride for Photocatalytic Conversion of Carbon Dioxide

**DOI:** 10.3390/polym11010146

**Published:** 2019-01-16

**Authors:** I-Hsiang Tseng, Yu-Min Sung, Po-Ya Chang, Chin-Yi Chen

**Affiliations:** 1 Department of Chemical Engineering, Feng Chia University, Taichung 40724, Taiwan; skycorn820312@gmail.com (Y.-M.S.); poyachang52@gmail.com (P.-Y.C.); 2 Department of Materials Science and Engineering, Feng Chia University, Taichung 40724, Taiwan; chencyi@fcu.edu.tw

**Keywords:** artificial photosynthesis, carbon dioxide, graphitic carbon nitride, titanium dioxide

## Abstract

Three types of graphitic carbon nitride (gCN) nanosheets were derived from direct thermal condensation of urea, melamine, and dicyandiamide, respectively. As the sample (uCN) synthesized from urea exhibited porous morphology and highest surface area among other gCN, anatase TiO_2_ nanoparticles were then in-situ deposited on uCN via solvothermal process without further calcination. The resultant Ti/uCN_x samples remained with higher surface area and exhibited visible-light activity. The derived band structure of each sample also confirmed its ability to photoreduce CO_2_. XPS results revealed surface compositions of each sample. Those functional groups governed adsorption of reactant, interfacial interaction, electron transfer rate, and consequently influenced the yield of products. Carbon monoxide and methanol were detected from LED-lamp illuminated samples under appropriate moisture content. Samples with higher ratio of terminal amine groups produced more CO. The presence of hydroxyl groups promoted the initial conversion of methanol. The obtained Ti/uCN_0.5 and Ti/uCN_1.5 samples exhibited better quantum efficiency toward CO_2_ conversion and demonstrated stability to consistently produce CO under cycling photoreaction.

## 1. Introduction

The polymeric semiconductor, graphitic carbon nitride (gCN), has attracted much attention due to its narrow and tunable bandgap for various photocatalytic applications [1,2,3,4,5]. Among them, artificial photosynthesis has been considered a challenging but promising and beneficial strategy to convert greenhouse gas CO_2_ into valuable products by using photocatalysts under sunlight [1,6,7,8]. Recently, researchers using gCN based photocatalytic system for photoreduction of CO_2_ have shown great progress [1,7,8,9,10,11,12,13,14,15,16,17,18,19]. To improve its photocatalytic activity, the morphology of gCN has been modified or various heterojunctions have been developed to increase surface area of gCN or to more effectively separate photoinduced charge carriers on gCN. TiO_2_ is the most popular photocatalyst for CO_2_ reduction. The presence of conductive graphitic network next to TiO_2_ would enhance electron–hole separation, increase the concentration of electron on the TiO_2_ surface, and even improve CO_2_ adsorption through π–π conjugation. The reaction of N–C=N with CO_2_ as carbamate confirmed that the gCN structure may accelerate the reduction of CO_2_ [20,21]. In addition, the product yield or selectivity was tunable by changing the relative ratio of gCN and TiO_2_ [16,17]. Even a relatively porous gCN with larger surface area showed higher photoactivity on CO_2_ conversion under visible light [18]. 

In this work, gCN was selected as the carbon-based substrate for TiO_2_ to evaluate its photocatalytic efficiency toward CO_2_ conversion under illumination by a 14-Watt LED light bulb. The anatase TiO_2_ nanoparticles were successfully synthesized via solvothermal process and deposited on gCN derived from different monomers. The effects of gCN chemical structures and relative TiO_2_ contents on the efficiency of CO_2_ conversion were comprehensively investigated.

## 2. Materials and Methods

### 2.1. Materials

Graphite powder (325 mesh), melamine (99%), dicyandiamide (99%) and titanium (IV) n-butoxide (TBOT, 99%) were purchased from Alfa Aesar (Lancashire, United Kingdom). Acetic acid (HAc, 99.7%), sulfuric acid (97%), hydrogen chloride (35%), and dihydrogen dioxide (30%) were provided from Showa (Saitama, Japan). n-Butanol (BtOH, 99.5%) from Scharlau (Barcelona, Spain) and urea from Uniregion Biotech (Miaoli, Taiwan) were used without further purification.

### 2.2. Synthesis of TiO_2_-Decorated gCN Nanosheets

Three types of graphitic carbon nitride, mCN, uCN, and dCN, were synthesized from the pyrolysis of melamine, urea, and dicyandiamide, respectively, from room temperature, at a rate of 6 °C/min, to 550 °C maintaining at 550 °C for further 3 h. According to the characterization results, an adequate amount (0.25–2.00 g) of uCN was selected and dispersed in n-butanol with the assistance of ultrasonication, followed by the addition of 4.27 mL titanium butoxide (TBOT) and adequate acetic acid. TBOT was then hydrolyzed by stoichiometric amounts of slowly released water from esterification of n-butanol and acetic acid. The well-mixed 50-mL solution was placed in a 100-mL Teflon-lined stainless-steel autoclave and maintained at 180 °C for 12 h. During the solvothermal process, the produced titanium hydroxide could react with the amine groups on uCN to anchor titania nanoparticles. After centrifugation, the obtained precipitate was washed with ethanol several times and followed by freeze-drying to acquire TiO_2_-decorated gCN. The sample name was denoted T-uCN-x, where x, ranging from 0.25 to 2, indicates the mass ratio of uCN to theoretical TiO_2_ mass converting from added TBOT amount. 

### 2.3. Characterization

The crystalline structure of synthesized nanosheets were determined by an X-ray diffractometer (XRD, D8SSS, BRUKER, Billerica, MA, USA) using Cu Kα radiation (λ = 0.154 nm) at an accelerating voltage of 40 kV and current of 30 mA to study the crystalline structure of powders. An X-ray photoelectron spectrometer (XPS, PHI5000, ULVAC-PHI, Kanagawa, Japan) was used to probe the surface composition of samples. The field emission scanning electron microscope (SEM, JSM-7401F, JEOL, Tokyo, Japan) was performed at an acceleration voltage of 15 kV to observe the morphology of nanoparticles. The field emission transmission electron microscope (TEM, JEM-2100F, JEOL, Tokyo, Japan) equipped with EDS (X-MAX, OXFORD INSTRUMENT, Abingdon, United Kingdom) was also applied at an acceleration voltage of 200 kV to observe the morphology of carbon nanosheets and the dispersion of TiO_2_. The UV-VIS diffuse reflectance spectra of powder samples were acquired on a spectrophotometer (F-7000, HITACHI, Tokyo, Japan) with an integrating sphere at room temperature. Thermogravimetric analysis (TGA) of each sample was carried out with a TGA-Q500 from TA INSTRUMENT (New Castle, DE, USA) at a heating rate of 20 °C/min under air. The photoluminescence (PL) of samples were measured on a fluorescence spectrophotometer (F-4500, HITACHI, Tokyo, Japan) using an excitation wavelength of 315 nm. A surface area and porosimetry analyzer (ASAP2020, MICROMERITICS, Norcross, GA, USA) was utilized to determine the BET surface area and pore size distribution of samples.

The photocatalytic reduction of CO_2_ was conducted in a batch reactor (100 mL) at ambient temperature. Powder sample (0.1 g) were dispersed on the bottom of a stainless-steel cylindrical reactor with the diameter of 12 cm and the height of 8 cm. A quartz window (3 cm in diameter) on the top of the reactor was designed for visible light illumination. According to manufacturer data, the luminous flux of the LED light bulb (14 W, PHILIPS, Taiwan) is 1400 lumens (lm). A luxometer (LT LUTRON, Taiwan) was utilized to measure the intensity of the visible light reaching the surface of catalysts in the reactor, which was 14,400 lux (14,400 lm/m^2^ or approximately 0.06 W). Another UVX radiometer (ANALYTIKJENA, Jena, Germany) equipped with UVC (254 nm) and UVA (365 nm) probes was also applied to measure UV intensity. Very weak UV intensity was detected by the probes, which was 2 μW/cm^2^ for UVC and 1.7 μW/cm^2^ for UVA. That is, the total photons (both visible and UV lights) reached the surface of catalysts were 0.02344 moles after 12 h illumination. Before illumination, the reactor was cleaned and placed in a vacuum oven at 40 °C for 1 h to remove gaseous impurity from the reactor. High purity CO_2_ (99.999%) was then purged through a water bubbler at room temperature into the reactor at a flowrate of 70 mL/min for 30 min. The outlet and inlet of the reactor were then closed and ready for the photoreaction. A gas chromatography (YL6500, YL INSTRUMENT, Gyeonggi-do, Korea) equipped with a highly sensitive pulsed-discharge helium ionization detector (PDHID, YL INSTRUMENT, Gyeonggi-do, Korea) was employed to analyze the products of photoreduction. The carrier gas was He, and two separation columns from Ohio Valley Specialty, including Porapack N for the detection of H_2_, O_2_, CO and CH_4_, and Molecular Sieve 5A for CO_2_, H_2_O and other C_1_–C_2_ hydrocarbons, were connected in series to simultaneously analyze all possible products from one injection. The volume of each injection was 1 mL of gas product withdrawn from the photoreactor by a gas-tight syringe.

## 3. Results and Discussion

**Figure 1:**
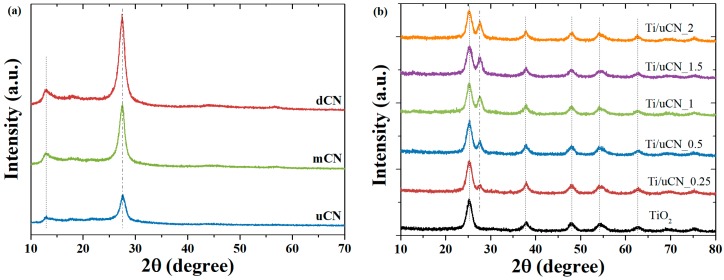
XRD patterns of: (**a**) gCN; and (**b**) Ti-uCN.

**Figure 2:**
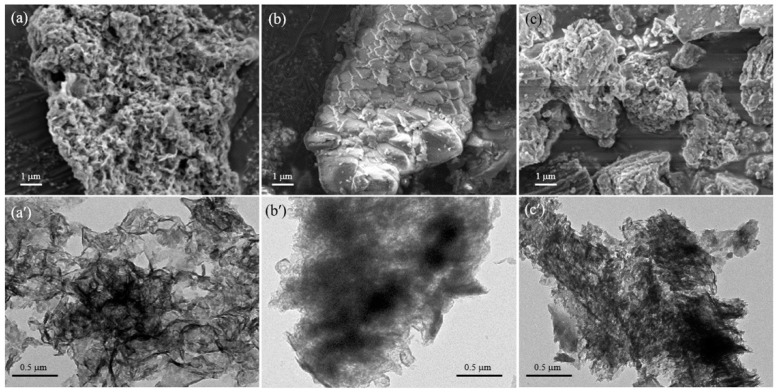
SEM and TEM images of: (**a**,**a′**) uCN; (**b**,**b′**) mCN; and (**c**,**c′**) dCN.

**Figure 3:**
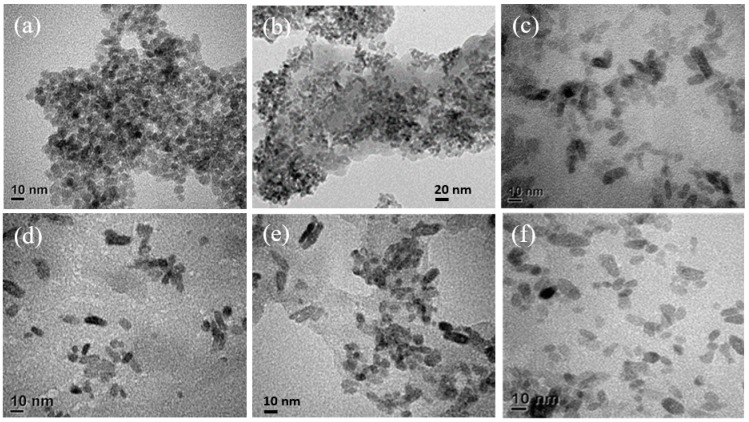
TEM images of: (**a**) TiO_2_; (**b**) Ti/uCN_0.25; (**c**) Ti/uCN_0.5; (**d**) Ti/uCN_1; (**e**) Ti/uCN_1.5; and (**f**) Ti/uCN_2.

The XRD patterns of three types of gCN and Ti-uCN nanosheets are shown in Figure 1. The characteristic (002) peak at around 27.5° is ascribed to the stacking of conjugated aromatic structure of gCN with the interlayer distance of 0.324 nm [2,5,19]. The weak one appeared at 13.0° was indexed as (100) plane, the in-planar tri-s-triazine units of gCN [22]. The difference in the position of (002) or (100) peaks between each gCN was negligible. During the deposition of gCN by the solvothermal process, the distance of the inter-planar stacking of (002) remained that the peaks at 27.5° were observed from all Ti/uCN-x samples. Besides, the characteristic anatase peaks, dot lines indicated in Figure 1b, confirmed the crystallinity of anatase from Ti/uCN-x. According to Scherrer’s equation, the crystalline size of anatase particles was around 10 nm. The decorated anatase TiO_2_ nanoparticles on each gCN nanosheets can also be observed from the following TEM images. Three gCN nanosheets derived from various precursors showed diverse morphology and surface area. Figure 2 displays the SEM and TEM images of each gCN. The porous morphology of uCN revealed from Figure 2a,a′ is consistent with its high surface area of 88 m^2^/g, which is the highest among others and literature value [22]. The presence of mesopores (~13 nm) within uCN enhanced the harvesting of light due to more frequent light-scattering [23]. Both mCN and dCN exhibited low surface area of 14 m^2^/g (Table 1). As uCN exhibited the highest surface area, uCN was selected to decorate TiO_2_. The BET surface area of TiO_2_ decorated uCN was as high as 170 m^2^/g, which is slightly lower than in-house TiO_2_ powders. The morphology of a series of TiO_2_-decorated uCN nanosheets could be observed from the high resolution TEM images (Figure 3). For comparison, the morphology of in-house TiO_2_ nanoparticles derived from identical process parameters except the addition of gCN is displayed in Figure 3a. The typical TiO_2_ nanoparticles were spherical and around 10 nm in diameter. As shown in Figure 3b, the surface of uCN was highly covered with TiO_2_ spheres with the diameter less than 10 nm as the mass ratio of uCN to TiO_2_ is 0.25, i.e., Ti/uCN_0.25. With the increase in uCN content, the shapes of TiO_2_ converted from sphere to rod, where the diameter remained less than 10 nm and the length varied with the uCN content. The axial growth of TiO_2_ particles was significant from samples containing more uCN. The curve layers presented in uCN (Figure 2a′) might be relevant to the formation of rod-like TiO_2_. 

**Figure 4:**
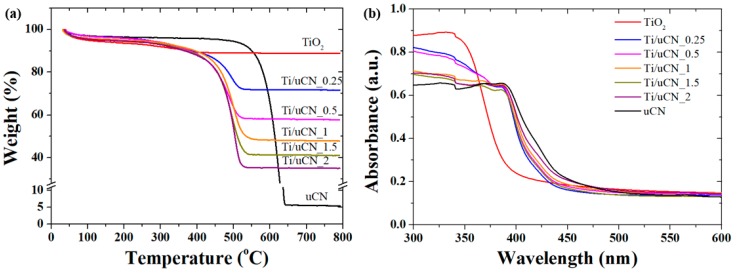

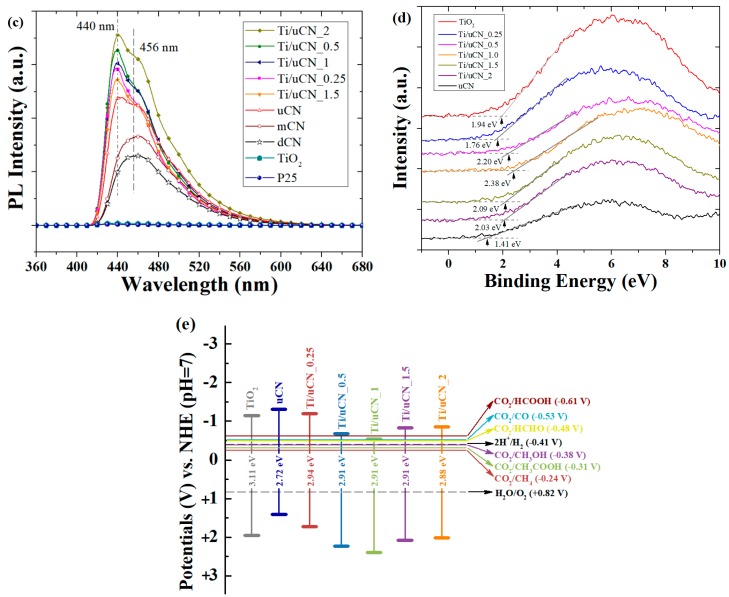
(**a**) Thermogravimetric curves; (**b**) UV-Vis spectra; and (**c**) photoluminescence spectra (Ex. λ = 315 nm); (**d**) XPS valence band spectra of uCN, TiO_2_ and Ti/uCN_x; and (**e**) schematic band edge position of samples with respect to redox potentials of species measured at pH 7.

The thermogravimetric curves of uCN, TiO_2_ and Ti/uCN_x in the air are shown in Figure 4a to evaluate the thermal stability of samples and estimate the content of titania on uCN. For uCN, the initial weight loss, which might be attributed to the loss of adsorbed water molecules, was less than 5% up to 550 °C, indicating its excellent thermal stability [24]. A significant decrease in weight was followed and ceased at 650 °C with the char yield of 5 wt %. With the presence of TiO_2_ nanoparticles on gCN, the thermal degradation of gCN occurred at a lower temperature of 450 °C, comparing to pure gCN, and completed at 550 °C. The char yield of Ti/uCN_x decreased with increasing x value as the char yield was an indicator of TiO_2_ content in each sample. Based on the residual weight of each curve, the relative gCN or TiO_2_ content in each sample was estimated and the results are listed in Table 1. The calculated values were similar to ideal ones, which were obtained by adding the precursor amounts.

The UV-vis diffuse reflectance spectra of TiO_2_, uCN, and a series of TiO_2_-decorated uCN nanosheets are depicted in Figure 4b. A significant red shift on the absorption edge was observed from Ti/uCN_x samples compared to reference TiO_2_ nanoparticles. The indirect bandgap of each sample was determined by Tauc plot [25] and the results are summarized in Table 1. The bandgap of reference in-house TiO_2_ and commercial P25 TiO_2_ was 3.2 eV and 3.1 eV, respectively, which is consistent to literature values. The red shift in bandgap energy of Ti/uCN_x was revealed, decreasing from 2.94 eV to 2.88 eV with increasing gCN contents. The intrinsic uCN exhibited a bandgap energy of 2.72 eV, which is slightly smaller than other gCN samples. It has been reported that better condensation degree of gCN-precursor led to stronger conjugative effect and consequently showed reduced bandgap and stronger PL signals [26]. The photoluminescence (PL) spectra of each gCN sample under the excitation at 315 nm at ambient condition are displayed in Figure 4c. The PL intensity of uCN was stronger than other gCN samples, indicating higher order of conjugated units from uCN. The PL wavelength at 456 nm is correlated to the free excitonic emission, as it is consistent to the determined band gap energy of 2.72 eV from UV-Vis spectrum of uCN [27]. The shorter wavelength emission may suggest the intra-transition of electrons from LUMO-π* state to the HOMO- state [27,28,29]. On the other hand, the tailing of PL in longer wavelength suggested the presence of structure detects. The difference in the shapes between three gCN samples suggested varied band structures of gCN [27]. The broad and intense PL emission in the visible range was observed for each Ti/uCN_x. In contrast, weak PL was observed from the reference in-house TiO_2_ and commercial P25 samples under the same excitation. Notably, despite lower uCN content, the intensity of PL emitted from Ti/uCN_x was stronger than that from uCN. The presence of TiO_2_ in uCN may promote the charge transfer from TiO_2_ to uCN, or the conjugation system was improved during TiO_2_ decoration process, and, consequently, more radiative relaxation occurred [27,29]. 

**Figure 5:**
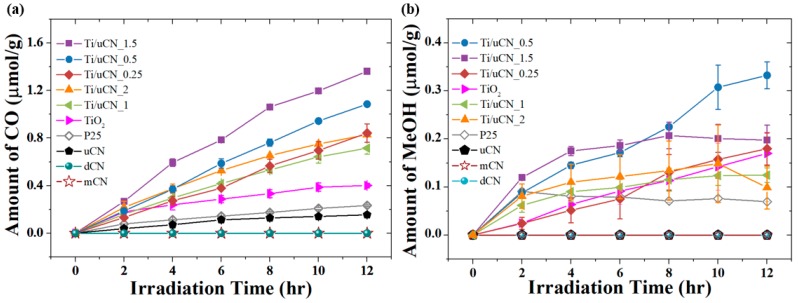
Time courses of (**a**) CO and (**b**) MeOH yields from each sample under LED illumination.

The valence band positions of each gCN and Ti/uCN_x samples were estimated by extrapolating a linear fit to the lower binding energy edge of the valence band photoemission to the baseline of XPS valence band (VB) spectra shown in Figure 4d. The VB edge potential was around 1.41 eV for uCN, 1.39 eV for mCN, and 1.14 eV for dCN, which are close to literature values [30,31]. Comparing to gCN, a more positive VB potential of 1.94 eV was obtained from in-house TiO_2_ sample. For Ti/uCN_x samples, their VB edge significantly varied with the composition and was in the range of 1.76–2.38 eV. According to estimated VB edge and bandgap energies, the conduction band (CB) band edge of each sample was estimated. Therefore, the band structure of each sample was plotted and CO_2_ reduction potentials with reference to NHE at pH 7 were designated (Figure 4e) [32,33]. A series of Ti/uCN_x samples showed more negative CB potentials than those of most CO_2_ reduction pathways, indicating their ability to reduce CO_2_. Notably, the oxidation capability of Ti/uCN_0.5, Ti/uCN_1, Ti/uCN_1.5, and Ti/uCN_2 was stronger than that of pure TiO_2_ based on their relative VB position. On the other hand, the reduction ability of those samples was in the opposite trend as the difference in bandgap energy between samples was relatively small. The ranking of the potential of VB or CB edge was not directly relevant to the following CO_2_ conversion yield (Figure 5). For example, Ti/uCN_0.25 exhibited highest reduction potential, however, weaker absorption of visible light (larger band gap) may contribute to its lower activity. In comparison to Ti/uCN_1.5 and Ti/uCN_2, which exhibited similar VB and CB edges, the low activity of Ti/uCN_2 may result from its low surface area. In summary, the decoration of TiO_2_ on uCN influenced the band edge and band gap energies. These factors combining with surface area, charge transfer mobility and surface functional groups contributed to its activity to convert CO_2_.

The ability of samples to convert CO_2_ under visible light illumination was evaluated by comparing their total yields of products. In this work, carbon monoxide (CO) was the main product and simultaneously trace amount of methanol (MeOH) was also obtained from samples. The time courses of CO and MeOH yields from each sample under LED illumination are plotted in Figure 5a,b, respectively. For pure gCN samples, only uCN could convert CO_2_ into CO under current experimental condition. After 10-h irradiation, in-house TiO_2_ produced 0.4 μmol/g CO, which is twice the yield from commercial P25, but levelled off upon further illumination. With the presence of uCN in TiO_2_, the increase in CO yield was observed from all Ti/uCN_x samples. Ti/uCN_1.5 exhibited the highest CO yield of 1.4 μmol/g after 12-h illumination, followed by Ti/uCN_0.5, which produced 1.1 μmol/g of CO. Notably, there were very small amounts of UVC and UVA lights reaching the surface of samples, as mentioned in Experimental Section, that even pure TiO_2_ could produce CO or MeOH in this work. On the other hand, Ti/uCN_0.5 showed higher selectivity to MeOH production than Ti/uCN_1.5 under the same reaction condition. The yield of MeOH was around 0.3 μmol/g from 12-h illuminated Ti/uCN_0.5. The production rate of MeOH from Ti/uCN_1.5 was highest during first 2-h illumination, however, the yield of MeOH levelled-off upon further illumination. Recall that Ti/uCN_1.5 emitted the weakest PL signal, therefore exhibiting better charge transfer toward the production of CO. In addition to Ti/uCN_1.5, other samples, Ti/uCN_1, Ti/uCN_2 and P25, showed similar phenomena as the shift from MeOH to other products occurred from those samples. Continuous production of MeOH was observed from in-house TiO_2_, Ti/uCN_0.25 and Ti/uCN_0.5, which contained more TiO_2_. As displayed in Figure 5, the standard deviation of MeOH yield was larger than that of CO yield. The reason is that three syringes were used to withdraw gas sample from the reactor at each data point to obtain an average value of product yield. The condensation of MeOH within syringe may occur during the waiting of GC analysis. In this work, trace amount of MeOH produced from Ti/uCN_x and reference TiO_2_ samples, but not from three gCN samples.

**Figure 6:**
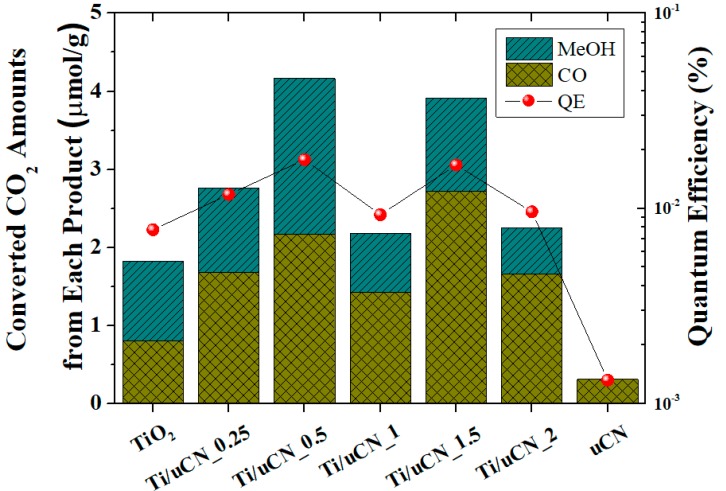
Converted CO_2_ amounts and quantum efficiency (QE) of each sample.

To easily compare current results with the literature, the quantum efficiency (QE) of each sample was estimated [34]. QE is defined as the total numbers of electrons required to convert CO_2_ into CO (two electrons) or MeOH (six electrons) divided by the incident photons at designated illumination period. As shown in Figure 6, the QE of each sample under 12-h LED illumination increased from 10^−3^% to 10^−2^% upon the combination of TiO_2_ with uCN. The sample Ti/uCN_1.5 exhibited the QE of 0.017% and the sample Ti/uCN_0.5 exhibited the highest QE of 0.018%, which is higher than other research groups using high-watt Xe lamps or doping with noble metals on gCN [19,35,36,37,38,39].

**Figure 7:**
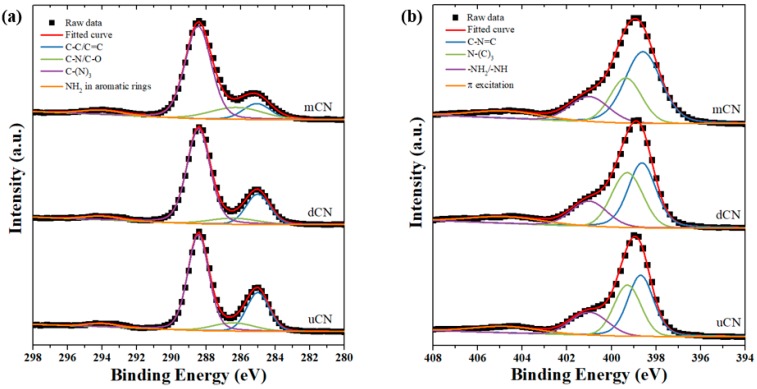
XPS spectra and deconvoluted curves in (**a**) C1s and (**b**) N1s region of gCN samples.

**Figure 8:**
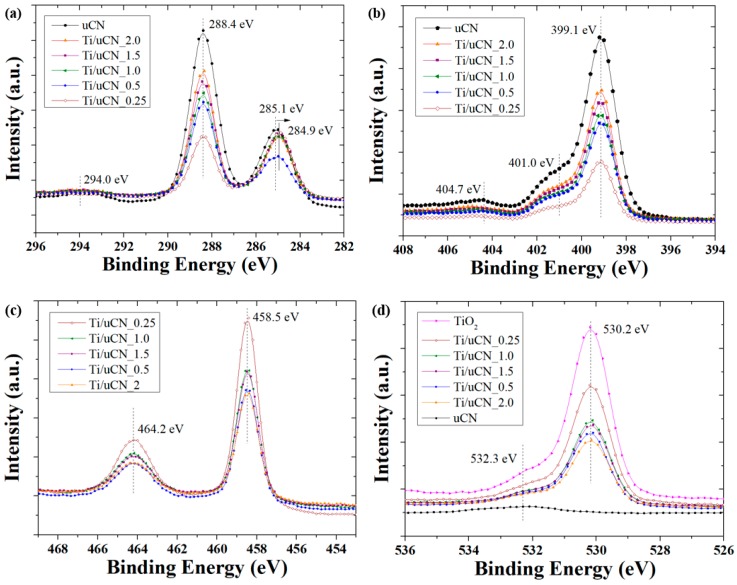
XPS spectra of uCN and Ti/uCN_x samples in: (**a**) C1s; (**b**) N1s; (**c**) Ti2p; and (**d**) O1s regions.

XPS spectra of a series of Ti/uCN_x samples were analyzed to correlate the surface functional groups of samples to their activity of CO_2_ conversion. The XPS C1s spectra of three gCN and their deconvoluted curves are plotted in Figure 7a. The C components centered at 285.1 eV were sp^2^ graphite structure and those at 286.4 eV were related to C–NH_2_ or C–O bonds, which might be attributed to the adsorption of H_2_O or CO_2_ [9,20,30]. The other peak displayed on 288.5 eV corresponded to the characteristic N=C–N_2_ or C–(N)_3_ bonds in tri-s-triazine units [5,24,30]. The XPS N1s spectra of each gCN sample, shown in Figure 7b, also revealed these characteristic nitrogen signals at 398.6 eV for C–N=C, 399.3 eV for N–(C)_3_, 401.0 eV for –NH_2_/=NH and 404.7 eV for π excitation [9,10,19,27,40]. With the presence of TiO_2_ on gCN, the XPS spectra of C1s, N1s, O2p and Ti2p core electrons are shown in Figure 8a–d. The characteristic tri-s-triazine units, featuring the peaks at 288.4 eV and 399.1 eV, were observed from all Ti/uCN_x samples and the peak intensity was consistent with the corresponding uCN amount, i.e. x value. The shift in the position of peaks corresponding to graphitic sp^2^ graphite structure was observed from all Ti/uCN_x samples, suggesting the existence of interaction between two phases [41,42]. However, the Ti–O–C bonds could not be obviously deconvoluted from C1s or Ti2p spectra, but from O1s spectra of Ti/uCN_x samples [25,43]. The XPS spectra in Ti2p region presented the dominant Ti–O bonds with the splitting of Ti2p electrons of 5.7 eV, further confirming the anatase phase of TiO_2_.

The surface compositions of C–, N– and O-containing functional groups were estimated by integrating each deconvoluted peak. The results are summarized in Table 2 and Table 3. Samples with higher ratio of terminal amine groups, –NH_2_ or =NH (401.0 eV), and Ti–O–C (533.3 eV) tended to higher CO yield as more CO_2_ adsorption and stronger interaction may improve their photocatalytic conversion [44,45,46]. Recently, the mechanism of CO formation from CO_2_ has been proposed [47]. The formation of carbamate is essential for the following production of CO. Two adjacent bare nitrogen atoms at the edge of gCN tend to attract protons and CO_2_, respectively, and then assist the activation of CO_2_ into carbamate. Therefore, the higher conversion efficiency achieved by samples with higher terminal amine groups may provide appropriate adsorption sites to active CO_2_ transfer into the intermediate of CO. On the other hand, the formation of MeOH requires more complex charge transfer steps and the presence of abundant hydrogen sources, e.g. water or hydrogen [45,48,49,50]. We also noticed that adequate amount of water in the photoreactor was essential to produce MeOH from CO_2_. In our case, MeOH was not the main product because we only provided small amounts of water vapors in the reactor. Therefore, the presence of more OH groups on catalysts will promote more adsorption of water molecules on the surface leading to higher MeOH yield. However, the produced MeOH may then act as sacrificial agent to promote the other reactions. That is, the decay in MeOH yield was attributed to further charge transfer and formation of other products.

**Figure 9:**
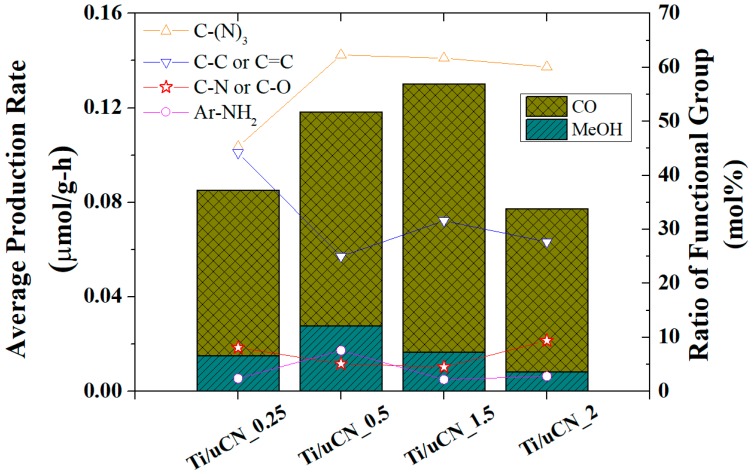
Relationship between initial (2 h) yield of products and surface ratio of carbon-contained functional groups of Ti/uCN_x samples.

In Figure 9, the average production rate, which is the accumulated yields from both CO and MeOH divided by the illumination time (12 h), of each sample is displayed in stacked columns and the relative composition of carbon-contained functional groups are symbols with guide lines. However, the product yield was not linearly relevant to the content of a specific functionality on each sample as the composition of surface functional groups were not the only factor governing the photoactivity of each sample. The ability to absorb visible light, the reduction potential, the surface area and the surface functional groups of samples attribute to the resultant activity or the selectivity of products. For example, Ti/uCN_0.25 exhibited highest reduction potential, weaker absorption of visible light (larger band gap), which might contribute to its lowest activity. In addition, the imperfection of tri-s-triazine units would inhibit the transfer of charge carriers; consequently, samples containing lower ratio of C–(N)_3_ groups or higher C–C/C=C groups exhibited lower activity. It elucidates again the low activity of Ti/uCN_0.25. The presence of Ti–O–C indicates the interaction between TiO_2_ and uCN. Therefore, samples containing more Ti–O–C suggest stronger interaction between two phases. For Ti/uCN_1, the lowest CB position combined with the lowest ratio of Ti–O–C component led to its lowest activity among other Ti/uCN_x. Both Ti/uCN_0.5 and Ti/uCN_1.5 have higher ratio of C-(N)_3_ groups, lower ratio of C–C/C=C groups, and relatively high content of Ti–O–C indicating more efficient charge transfer within their matrix. In comparison of Ti/uCN_1.5 and Ti/uCN_2, which exhibited similar VB and CB edges and similar surface composition, the low activity of Ti/uCN_2 may result from the significant reduce in surface area. The higher methanol production rate from Ti/uCN_0.5 might be attributed to its higher content of terminal NH_2_ groups than Ti/uCN_1.5. The initially produced methanol from Ti/uCN_1.5 converted into other products such as CO upon further illumination.

**Figure 10:**
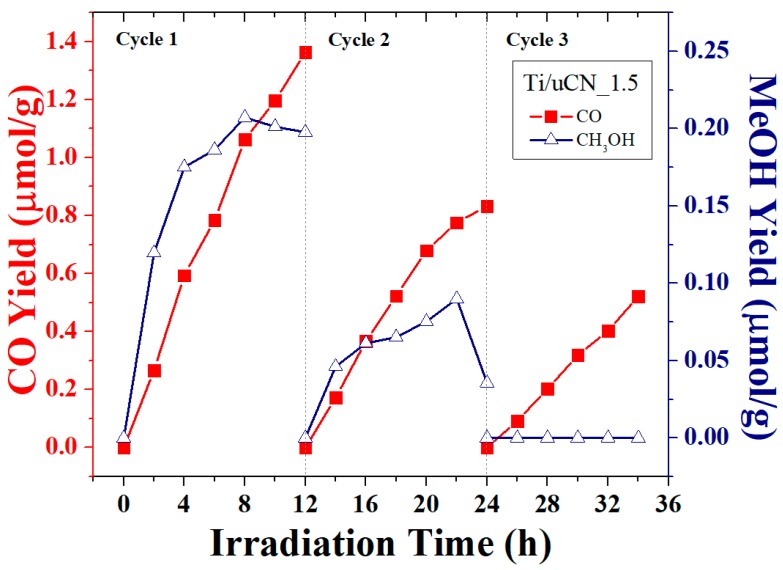
CO and MeOH yield from Ti/uCN_1.5 under three reaction cycles.

**Figure 11:**
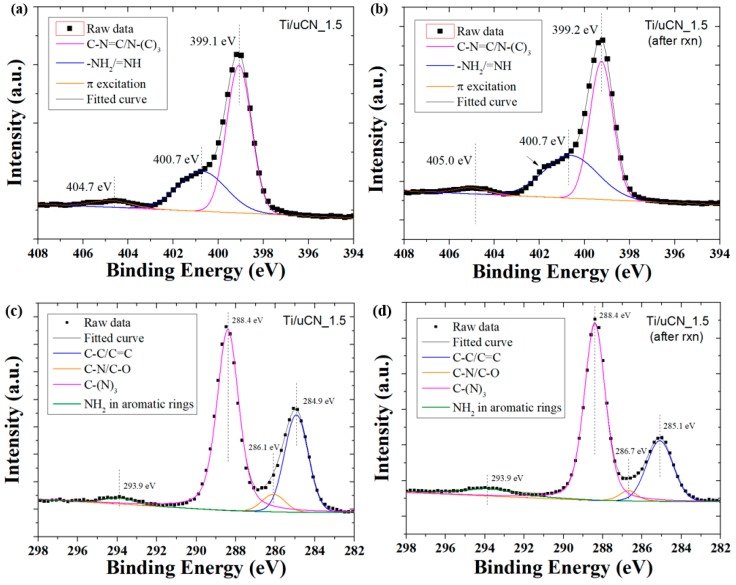

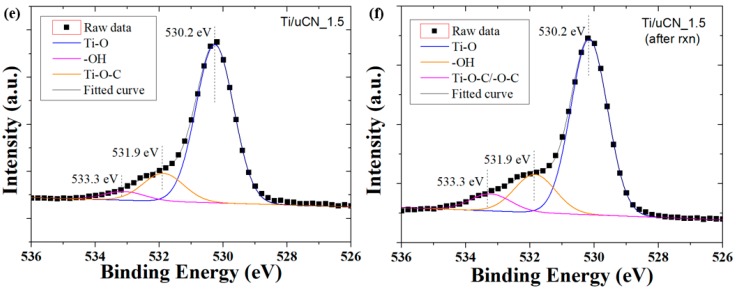
Deconvoluted XPS spectra in: (**a**,**b**) N1s; (**c**,**d**) C1s; and (**e**,**f**) O1s regions from Ti/uCN_1.5 (**a**,**c**,**e**) before and (**b**,**d**,**f**) after photoreduction.

The cycling test of each sample was conducted to investigate its stability. Figure 10 plots the ability of Ti/uCN_1.5 to convert CO_2_ into CO or MeOH under each reaction cycle. Although the decrease in CO yield was revealed after each reaction cycle, a stable production rate of 0.05 μmol/g-h was achieved at Cycle 3. The XPS spectra together with the deconvoluted curves of Ti/uCN_1.5 before and after photoreaction are shown in Figure 11. The surface components of this sample after photoreduction are summarized in Table 2 and Table 3. The slight shift in binding energy of graphitic groups and significant decrease in the amount of C–C/C=C groups were revealed from Ti/uCN_1.5 sample after reaction. Moreover, the increase in structure defects (around 400.7 eV) and the coverage of active sites (increasing impurity at 533.3 eV) on samples may result in the decrease in photoactivity. Notably, after each reaction cycle, the sample within the reactor was merely placed in a vacuum oven at 80 °C for 2 h. Further activation process was required to retain the intrinsic photoactivity of Ti/uCN_1.5.

## 4. Conclusions

In this work, anatase TiO_2_ nanoparticles were successfully synthesized under solvothermal process and simultaneously deposited on uCN. The uCN containing TiO_2_ samples exhibited high surface area and sufficient reduction potential to reduce gaseous CO_2_ under illumination of 14-Watt LED light bulb without using sacrificial agents. The composition of surface functionality on each sample was not the only factor governing the photoactivity of each sample. The ability to absorb visible light, the reduction potential, the surface area and the surface functional groups of samples all attributed to the resultant activity or the selectivity of products. The obtained Ti/uCN_0.5 and Ti/uCN_1.5 samples exhibited better quantum efficiency toward CO_2_ conversion and would consistently produce CO over cycling test.

## Figures and Tables

**Table 1:** Characteristics of Ti/uCN_x, TiO_2_ and uCN samples.

Sample Code	Char Yield (%)	Mass Ratio (uCN/TiO_2_)		BET Surface Area (m^2^/g)	Band Gap ^c^ (eV)
		Ideal ^a^	Actual ^b^		
TiO_2_	88.88	-	-	193	3.11
Ti/uCN_0.25	71.65	0.25	0.28	170	2.94
Ti/uCN_0.5	57.73	0.50	0.61	-	2.91
Ti/uCN_1	47.73	1.00	0.99	-	2.91
Ti/uCN_1.5	41.08	1.50	1.36	156	2.91
Ti/uCN_2	35.10	2.00	1.82	44	2.88
uCN	5.34	-	-	88	2.72
dCN	-	-	-	14	2.78
mCN	-	-	-	14	2.75

^a^ Mass ratio of added gCN to resultant TiO_2_ from added TBOT (4.27 mL). ^b^ Calculated from TGA char yield. ^c^ Estimated from Tauc Plot. The band gap of commercial TiO_2_, P25, is 3.24 eV.

**Table 2:** Surface composition of carbon-contained functional groups based on XPS C1s fitting results.

Sample Code	C1s Composition (%)			
	C–C/C=C (285.1 eV) ^a^	C–N/C–O (286.4 eV) ^a^	C–(N)_3_ (288.4 eV) ^a^	NH_2_ in Aromatic Rings (293.9 eV) ^a^
Ti/uCN_0.25	44.2	8.1	45.3	2.4
Ti/uCN_0.5	25.0	5.1	62.3	7.6
Ti/uCN_1	31.9	4.9	58.2	5.0
Ti/uCN_1.5	31.6	4.5	61.7	2.2
Ti/uCN_1.5 ^b^	25.9	3.4	67.8	2.6
Ti/uCN_2	27.7	9.4	60.1	2.8
uCN	25.4	9.5	62.2	2.9
dCN	20.5	8.0	68.0	3.5
mCN	11.7	15.9	68.0	4.4

^a^ Approximate peak center position. ^b^ After photoreaction (Cycle 2).

**Table 3:** Surface composition of nitrogen-contained and oxygen-contained functional groups based on XPS N1s or O1s fitting results.

Sample Code	N1s Composition (%)			O1s Composition (%)		
	C–N=C/N–(C)_3_ (399.1 eV) ^a^	–NH_2_/=NH (401.0 eV) ^a^	π Excitation (404.7 eV) ^a^	Ti–O (530.2 eV) ^a^	–OH (531.9 eV) ^a^	Ti–O–C (533.3 eV) ^a^
Ti/uCN_0.25	54.2	41.7	4.0	81.7	14.5	3.8
Ti/uCN_0.5	64.9	30.5	4.6	77.6	16.7	5.7
Ti/uCN_1	63.8	31.8	4.4	81.5	15.4	3.1
Ti/uCN_1.5	62.7	32.8	4.5	80.0	15.3	4.8
Ti/uCN_1.5 ^b^	56.3	40.2	3.5	74.1	17.9	8.0
Ti/uCN_2	69.7	25.0	5.4	78.4	15.8	5.8
uCN	68.1	26.6	5.2	-	-	-
dCN	65.2	26.7	8.1	-	-	-
mCN	76.4	14.0	9.5	-	-	-

^a^ Approximate position of deconvoluted peak. ^b^ After photoreaction (Cycle 2).

## References

[1] Lu L.H., Lv Z.Z., Si Y.J., Liu M.Y., Zhang S. (2018). Recent progress on band and surface engineering of graphitic carbon nitride for artificial photosynthesis. Appl. Surf. Sci..

[2] Wang X., Maeda K., Thomas A., Takanabe K., Xin G., Carlsson J.M., Domen K., Antonietti M. (2009). A metal-free polymeric photocatalyst for hydrogen production from water under visible light. Nat. Mater..

[3] Liu G., Niu P., Sun C.H., Smith S.C., Chen Z.G., Lu G.Q., Cheng H.M. (2010). Unique electronic structure induced high photoreactivity of sulfur-doped graphitic C_3_N_4_. J. Am. Chem. Soc..

[4] Dong G.P., Zhang Y.H., Pan Q.W., Qiu J.R. (2014). A fantastic graphitic carbon nitride (g-C_3_N_4_) material: Electronic structure, photocatalytic and photoelectronic properties. J. Photochem. Photobiol. C Photochem. Rev..

[5] Li K., Gao S.M., Wang Q.Y., Xu H., Wang Z.Y., Huang B.B., Dai Y., Lu J. (2015). In-situ-reduced synthesis of Ti^3+^ self-doped TiO_2_/g-C_3_N_4_ heterojunctions with high photocatalytic performance under led light irradiation. ACS Appl. Mater. Interfaces.

[6] Tseng I.H., Chang W.C., Wu J.C.S. (2002). Photoreduction of CO_2_ using sol-gel derived titania and titania-supported copper catalysts. Appl. Catal. B Environ..

[7] Chang P.Y., Tseng I.H. (2018). Photocatalytic conversion of gas phase carbon dioxide by graphitic carbon nitride decorated with cuprous oxide with various morphologies. J. CO_2_ Util..

[8] Ohno T., Murakami N., Koyanagi T., Yang Y. (2014). Photocatalytic reduction of CO_2_ over a hybrid photocatalyst composed of WO_3_ and graphitic carbon nitride (g-C_3_N_4_) under visible light. J. CO_2_ Util..

[9] Sun Z.X., Wang H.Q., Wu Z.B.A., Wang L.Z. (2018). G-C_3_N_4_ based composite photocatalysts for photocatalytic CO_2_ reduction. Catal. Today.

[10] Shi G.D., Yang L., Liu Z.W., Chen X., Zhou J.Q., Yu Y. (2018). Photocatalytic reduction of CO_2_ to CO over copper decorated g-c3n4 nanosheets with enhanced yield and selectivity. Appl. Surf. Sci..

[11] Jiang Z.F., Wan W.M., Li H.M., Yuan S.Q., Zhao H.J., Wong P.K. (2018). A hierarchical z-scheme alpha-Fe_2_O_3_/g-C_3_N_4_ hybrid for enhanced photocatalytic CO_2_ reduction. Adv. Mater..

[12] Xia P.F., Zhu B.C., Yu J.G., Cao S.W., Jaroniec M. (2017). Ultra-thin nanosheet assemblies of graphitic carbon nitride for enhanced photocatalytic CO_2_ reduction. J. Mater. Chem. A.

[13] Tahir B., Tahir M., Amin N.A.S. (2017). Photo-induced CO_2_ reduction by Ch4/H_2_O to fuels over Cu-modified g-C_3_N_4_ nanorods under simulated solar energy. Appl. Surf. Sci..

[14] Zhang L.H., Jin Z.Y., Lu H., Lin T.Q., Ruan S.C., Zhao X.S., Zeng Y.J. (2018). Improving the visible-light photocatalytic activity of graphitic carbon nitride by carbon black doping. ACS Omega.

[15] Qin J., Wang S., Ren H., Hou Y., Wang X. (2015). Photocatalytic reduction of CO_2_ by graphitic carbon nitride polymers derived from urea and barbituric acid. Appl. Catal. B Environ..

[16] Dai K., Lu L.H., Liang C.H., Liu Q., Zhu G.P. (2014). Heterojunction of facet coupled g-C_3_N_4_/surface-fluorinated TiO_2_ nanosheets for organic pollutants degradation under visible led light irradiation. Appl. Catal. B Environ..

[17] Zhou S., Liu Y., Li J.M., Wang Y.J., Jiang G.Y., Zhao Z., Wang D.X., Duan A.J., Liu J., Wei Y.C. (2014). Facile in situ synthesis of graphitic carbon nitride (g-C_3_N_4_)-n-TiO_2_ heterojunction as an efficient photocatalyst for the selective photoreduction of CO_2_ to CO. Appl. Catal. B Environ..

[18] Mao J., Peng T.Y., Zhang X.H., Li K., Ye L.Q., Zan L. (2013). Effect of graphitic carbon nitride microstructures on the activity and selectivity of photocatalytic CO_2_ reduction under visible light. Catal. Sci. Technol..

[19] Yu J.G., Wang K., Xiao W., Cheng B. (2014). Photocatalytic reduction of CO_2_ into hydrocarbon solar fuels over g-C_3_N_4_-pt nanocomposite photocatalysts. Phys. Chem. Chem. Phys..

[20] Thomas A., Fischer A., Goettmann F., Antonietti M., Muller J.O., Schlogl R., Carlsson J.M. (2008). Graphitic carbon nitride materials: Variation of structure and morphology and their use as metal-free catalysts. J. Mater. Chem..

[21] Goettmann F., Thomas A., Antonietti M. (2007). Metal-free activation CO_2_ by mesoporous graphitic carbon nitride. Angew. Chem. Int. Ed..

[22] Zhu B.C., Xia P.F., Ho W.K., Yu J.G. (2015). Isoelectric point and adsorption activity of porous g-C_3_N_4_. Appl. Surf. Sci..

[23] Tang H., Chang S., Jiang L., Tang G., Liang W. (2016). Novel spindle-shaped nanoporous TiO_2_ coupled graphitic g-C_3_N_4_ nanosheets with enhanced visible-light photocatalytic activity. Ceram. Int..

[24] Bahuguna A., Choudhary P., Chhabra T., Krishnan V. (2018). Ammonia-doped polyaniline-graphitic carbon nitride nanocomposite as a heterogeneous green catalyst for synthesis of indole-substituted 4h-chromenes. ACS Omega.

[25] Tseng I.H., Sung Y.-M., Chang P.-Y., Lin S.-W. (2017). Photocatalytic performance of titania nanosheets templated by graphene oxide. J. Photochem. Photobiol. A Chem..

[26] Zhang Y.W., Liu J.H., Wu G., Chen W. (2012). Porous graphitic carbon nitride synthesized via direct polymerization of urea for efficient sunlight-driven photocatalytic hydrogen production. Nanoscale.

[27] Choudhury B., Paul K.K., Sanyal D., Hazarika A., Giri P.K. (2018). Evolution of nitrogen-related defects in graphitic carbon nitride nanosheets probed by positron annihilation and photoluminescence spectroscopy. J. Phys. Chem. C.

[28] Das D., Banerjee D., Pahari D., Ghorai U.K., Sarkar S., Das N.S., Chattopadhyay K.K. (2017). Defect induced tuning of photoluminescence property in graphitic carbon nitride nanosheets through synthesis conditions. J. Lumines..

[29] Praus P., Svoboda L., Ritz M., Troppova I., Sihor M., Koci K. (2017). Graphitic carbon nitride: Synthesis, characterization and photocatalytic decomposition of nitrous oxide. Mater. Chem. Phys..

[30] Kang Y.Y., Yang Y.Q., Yin L.C., Kang X.D., Liu G., Cheng H.M. (2015). An amorphous carbon nitride photocatalyst with greatly extended visible-light-responsive range for photocatalytic hydrogen generation. Adv. Mater..

[31] Guo S.E., Deng Z.P., Li M.X., Jiang B.J., Tian C.G., Pan Q.J., Fu H.G. (2016). Phosphorus-doped carbon nitride tubes with a layered micro-nanostructure for enhanced visible-light photocatalytic hydrogen evolution. Angew. Chem. Int. Ed..

[32] Shen M., Zhang L.X., Shi J.L. (2018). Converting CO_2_ into fuels by graphitic carbon nitride-based photocatalysts. Nanotechnology.

[33] Indrakanti V.P., Kubicki J.D., Schobert H.H. (2009). Photoinduced activation of CO_2_ on ti-based heterogeneous catalysts: Current state, chemical physics-based insights and outlook. Energy Environ. Sci..

[34] Xiong Z., Kuang C.C., Lin K.Y., Lei Z., Chen X.X., Gong B.G., Yang J.P., Zhao Y.C., Zhang J.Y., Xia B.Y. (2018). Enhanced CO_2_ photocatalytic reduction through simultaneously accelerated H_2_ evolution and CO_2_ hydrogenation in a twin photoreactor. J. CO_2_ Util..

[35] Yu W.L., Xu D.F., Peng T.Y. (2015). Enhanced photocatalytic activity of g-C_3_N_4_ for selective CO_2_ reduction to CH_3_OH via facile coupling of ZnO: A direct z-scheme mechanism. J. Mater. Chem. A.

[36] He Y.M., Zhang L.H., Fan M.H., Wang X.X., Walbridge M.L., Nong Q.Y., Wu Y., Zhao L.H. (2015). Z-scheme SnO_2_-x/g-C_3_N_4_ composite as an efficient photocatalyst for dye degradation and photocatalytic CO_2_ reduction. Sol. Energy Mater. Sol. Cells.

[37] Huang Y., Fu M., He T. (2015). Synthesis of g-C_3_N_4_/BiVO4 nanocomposite photocatalyst and its application in photocatalytic reduction of CO_2_. Acta Phys. Chim. Sin..

[38] Zhang X.J., Wang L., Du Q.C., Wang Z.Y., Ma S.G., Yu M. (2016). Photocatalytic CO_2_ reduction over B_4_C/C_3_N_4_ with internal electric field under visible light irradiation. J. Colloid Interface Sci..

[39] Bai Y., Chen T., Wang P.Q., Wang L., Ye L.Q., Shi X., Bai W. (2016). Size-dependent role of gold in g-C_3_N_4_/BiOBr/Au system for photocatalytic CO_2_ reduction and dye degradation. Sol. Energy Mater. Sol. Cells.

[40] Bian J.C., Huang C., Zhang R.Q. (2016). Graphitic carbon nitride film: An emerging star for catalytic and optoelectronic applications. ChemSusChem.

[41] Zhang Y.J., Schnepp Z., Cao J.Y., Ouyang S.X., Li Y., Ye J.H., Liu S.Q. (2013). Biopolymer-activated graphitic carbon nitride towards a sustainable photocathode material. Sci. Rep..

[42] Tan L.H., Xu J.H., Li S.Y., Li D.N., Dai Y.M., Kou B., Chen Y. (2017). Direct growth of cuo nanorods on graphitic carbon nitride with synergistic effect on thermal decomposition of ammonium perchlorate. Materials.

[43] Fu Y.Q., Du H.J., Zhang S., Huang W.M. (2005). Xps characterization of surface and interfacial structure of sputtered tini films on si substrate. Mater. Sci. Eng. A.

[44] Peng H.-L., Zhong F.-Y., Zhang J.-B., Zhang J.-Y., Wu P.-K., Huang K., Fan J.-P., Jiang L.-L. (2018). Graphitic carbon nitride functionalized with polyethylenimine for highly effective capture of carbon dioxide. Ind. Eng. Chem. Res..

[45] Fu J.W., Zhu B.C., Jiang C.J., Cheng B., You W., Yu J.G. (2017). Hierarchical porous o-doped g-C_3_N_4_ with enhanced photocatalytic CO_2_ reduction activity. Small.

[46] Sun Z., Wang S., Li Q., Lyu M., Butburee T., Luo B., Wang H., Fischer J.M.T.A., Zhang C., Wu Z. (2017). Enriching CO_2_ activation sites on graphitic carbon nitride with simultaneous introduction of electron-transfer promoters for superior photocatalytic CO_2_-to-fuel conversion. Adv. Sustain. Syst..

[47] Wu H.-Z., Bandaru S., Huang X.-L., Liu J., Li L., Wang Z.-L. (2018). Theoretical insight into the mechanism of photoreduction CO_2_ to CO by graphitic carbon nitride. Phys. Chem. Chem. Phys..

[48] Gondal M.A., Lais A., Dastageer M.A., Yang D., Shen K., Chang X. (2017). Photocatalytic conversion of CO_2_ into methanol using graphitic carbon nitride under solar, UV laser and broadband radiations. Int. J. Energy Res..

[49] Sarkar A., Gracia-Espino E., Wagberg T., Shchukarev A., Mohl M., Rautio A.R., Pitkanen O., Sharifi T., Kordas K., Mikkola J.P. (2016). Photocatalytic reduction of CO_2_ with H_2_O over modified TiO_2_ nanofibers: Understanding the reduction pathway. Nano Res..

[50] Wang B.Y., Chen W., Song Y.F., Li G.H., Wei W., Fang J.H., Sun Y.H. (2018). Recent progress in the photocatalytic reduction of aqueous carbon dioxide. Catal. Today.

